# Exploring measurement biases associated with esophageal Doppler monitoring in critically ill patients in intensive care unit

**DOI:** 10.4103/1817-1737.36548

**Published:** 2007

**Authors:** Peter S. Stawicki, Benjamin Braslow, Vicente H. Gracias

**Affiliations:** *Department of Surgery, Division of Traumatology and Surgical Critical Care, University of Pennsylvania School of Medicine, 3440 Market Street, 1^st^ Floor, Philadelphia, PA 19104-3335, USA*

**Keywords:** Critically ill patients, esophageal echo-Doppler, measurement bias, ventilated patients

## Abstract

**BACKGROUND:**

Esophageal Doppler monitoring (EDM) is utilized in numerous clinical settings. This study examines the relationship between pulmonary artery catheter (PAC) and EDM-derived hemodynamic parameters, concentrating on gender- and age-related EDM measurement biases.

**MATERIALS AND METHODS:**

Prospective study of EDM use in ventilated surgical ICU patients. Parameters examined included demographics, diagnosis, resuscitation endpoints, cardiac output (CO) and stroke volume from both devices, number of personnel and time needed to place equipment, time to data acquisition, duration of use, complications of placement.

**RESULTS:**

Fifteen patients (11 men, 4 women, mean age 47 years) were included. Most common diagnoses included trauma (7/15) and sepsis (4/15). Insertion time and time to data acquisition were shorter for EDM than for PAC (*P*<0.001). The EDM required an average of 1.1 persons to place (2.4 for PAC, *P*=0.002). Mean EDM utilization time was 12.4 h. There was a fair CO correlation between EDM and PAC (r = 0.647, *P*<0.001). Overall, the EDM underestimated CO relative to PAC (bias -1.42 ± 2.08, 95% CI: -5.58-2.74), with more underestimation in women (mean bias difference of -1.16, *P*<0.001). No significant age-related measurement bias differences between PAC and EDM were noted. Significant reductions in lactate and norepinephrine requirement were noted following EDM monitoring periods.

**CONCLUSIONS:**

This study found that the EDM significantly underestimated cardiac output in women when compared to PAC. Clinicians should be aware of this measurement bias when making therapeutic decision based on EDM data. Significant reductions in lactate and norepinephrine requirement during EDM monitoring periods support the clinical usefulness of EDM technology.

Esophageal Doppler monitoring (EDM) technology continues to evolve. Its potential usefulness has been demonstrated by prospective, randomized, controlled trials.[1,2] It has been utilized in numerous clinical settings, with reports from obstetric, trauma, pediatric, colon-rectal, intensive care unit, cardiac surgery organ donation and emergency department literature.[3–8] The purpose of this study is to prospectively determine the correlation between continuous cardiac output (CCO) pulmonary artery catheter (PAC)-derived and EDM-derived hemodynamic parameters and to examine EDM-guided resuscitation trends in critically ill surgical intensive care unit (SICU) patients. In addition, based on previously published data,[7] an examination of the relationship between EDM and PAC has been carried out with regards to patient gender- and age-related measurement bias.

## Materials and Methods

After Institutional Board Review approval, a prospective comparison study of the esophageal Doppler monitor (Hemosonic 100™, Arrow International, Reading, Pennsylvania, USA) and the continuous cardiac output (CCO) pulmonary artery catheter (PAC) was performed. All patients had a CCO-PAC and the esophageal echo-Doppler monitor (EDM) present simultaneously during intensive care unit (ICU) resuscitations. Measurements from both modalities of monitoring were recorded and entered into a computerized database, at least on an hourly basis. Patient exclusion criteria included age less than 18 years, death within the first 24 h of admission to the ICU, absence of PAC, contraindication to EDM placement as defined in previous report.[9] Included were adult patients (≥18 years old) who underwent pulmonary artery catheter placement during their ICU resuscitations and had no contraindications to EDM placement.

Clinical information collected included (a) patient demographics; (b) clinical diagnosis; (c) resuscitation-related parameters - lactic acid level and vasopressor requirement; (d) traditional vital signs; (e) analogous hemodynamic data from both monitoring modalities - cardiac output, stroke volume; (f) monitoring device placement characteristics - placement time, average number of personnel needed to place equipment, time to obtain first set of measurements, duration of EDM use; and (g) complications related to monitoring equipment placement.

In this study, the EDM was utilized episodically, based on the availability of critical care personnel trained in EDM use. While in place, the EDM was used as the primary determinant of therapeutic resuscitative interventions. During non-EDM monitoring periods, the PAC was used to guide patient resuscitations. Endpoints of resuscitation measurements of serum lactic acid level were compared at the beginning and at the end of each EDM period to document clinical efficacy of the resuscitation during the monitoring period. In addition, vasopressor requirement (norepinephrine and neosynephrine) was recorded at the beginning and at the end of each EDM monitoring period. Because of the observational nature of this study, no comparisons were made between EDM- and PAC-directed patient resuscitations.

Based on previously published data,[7] which demonstrated possible gender- and age-based measurement biases related to the EDM technology, specific comparisons between the CCO-PAC and the EDM parameters were performed with regards to patient age (<40 years versus ≥40 years) and patient gender. These comparisons included calculations of the coefficient of correlation as well as the calculation of differences in bias between the two methods with regards to the parameters of patient gender and age.

Statistical methods included Fisher's exact test for categorical variables, Wilcoxon signed ranks test and Student's t-test for continuous data; and coefficient of correlation, when required. Comparisons between the PAC- and EDM-derived parameters were carried out using the Bland-Altman bias plot methodology. Statistical significance was set at alpha = 0.05.

## Results

A total of 15 patients were studied from January 2000 to November 2002. There were 11 men and 4 women. Mean patient age was 47 ± 23 years (median 50, age range of 18-89). There was no statistically significant age difference between men (45 y/o) and women (55 y/o, P = NS). The mean APACHE II score for this group of patients was 18.8 ± 3.65 (median 18.5, range 14-25). Clinical diagnoses in this group of patients included multiple trauma (7/15), sepsis (4/15), abdominal compartment syndrome (2/15) and severe traumatic brain injury (2/15).

The mean insertion time for the EDM was 9.1 min (range 2-16 min). Mean time for PAC insertion was 25 min (range 7-45 min). This represented a significant difference in insertion times (*P*<0.001). The mean time from start of procedure to data acquisition for the EDM was 12 min (range 6-21 min). The mean time from beginning of PAC insertion to data acquisition was 60 min (range 25-110 min). This also represents a statistically significant difference (*P*<0.001). No clinically significant measurement failures (i.e. persistent loss of signal or need for equipment exchange) were noted for either of the monitoring modalities.

The mean number of health care personnel required to place EDM was 1.1 per insertion (range 1-2 persons). For the PAC, the mean number of personnel required was 2.4 (range 2-3 persons). The difference of 1.3 persons necessary for equipment insertion was statistically significant (*P*=0.002). Overall, the EDM was utilized for a mean duration of 12.4 ± 6.84 h per patient, with a median duration of 12.5 h and utilization time range between 3 and 21.5 h.

The cost per each EDM use was approximately $100, while the cost of each PAC placement was $400. The cost associated with each PAC placement included the cost of (a) the introducer catheter, (b) the pulmonary artery catheter and (c) the developed chest radiogram required after catheter placement. There were no complications related to EDM placement. One patient (6.7%) had an inadvertent carotid artery needle stick during PAC placement, which was promptly recognized and no clinical sequelae were noted.

**Figure 1:**
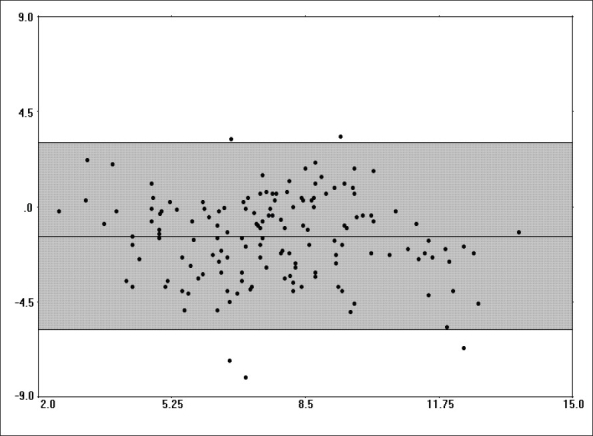
Bias plot comparing cardiac output measurements from continuous cardiac output PAC and EDM in the overall patient sample. The mean of the two methods can be seen on the X-axis. The difference between the two methods can be seen on the Y-axis. The shaded area indicates the 95% confidence interval (CI). Note that the overall bias is −1.42 ± 2.08, 95% CI −5.58 to 2.74.

**Figure 2:**
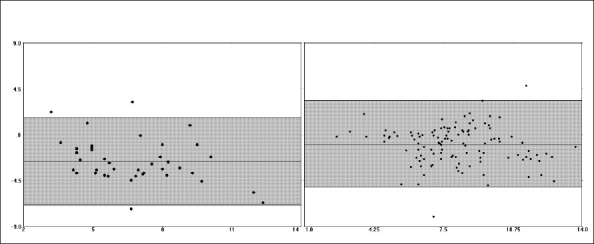
Bias plot comparing cardiac output measurements from continuous cardiac output PAC and EDM in women (left) and men (right). The mean of the two methods can be seen on the X-axis. The difference between the two methods can be seen on the Y-axis. The shaded area indicates the 95% confidence interval (CI). Note the significantly greater negative measurement bias noted in women - Table 1 for exact values.

**Figure 3:**
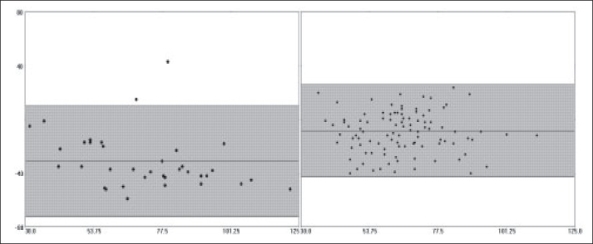
Bias plot comparing stroke volume measurements from continuous cardiac output PAC and EDM in women (left) and men (right). The mean of the two methods can be seen on the X-axis. The difference between the two methods can be seen on the Y-axis. The shaded area indicates the 95% confidence interval (CI). Note the significantly greater negative measurement bias noted in women - Table 1 for exact values.

The comparison of PAC and EDM data demonstrated that there was a fair amount of correlation between the two methods with regards to cardiac output measurements (r = 0.647, r^2^ = 0.418, *P*<0.001). No clinically significant correlations were noted between EDM-derived maximum acceleration (Acc) and pulmonary artery pressures (mean, systolic, diastolic) or PAC-derived stroke volume (all, r < 0.50, r^2^ < 0.20). The EDM tended to underestimate the cardiac output relative to the PAC in the overall patient sample (bias -1.42 ± 2.08, 95% CI -5.58-2.74, [Figure 1]). The underestimation of cardiac output was more pronounced in women than in men, with the difference in bias being statistically significant (mean difference of -1.16, 95% CI of difference -2.33 to -0.902, *P*<0.001) [Figure 2, Table 1]. Similar difference in bias was noted when examining stroke volume measurements by the two methods [Table 1, Figure 3]. No significant differences in bias were noted between PAC and EDM measurements when comparing patients who were younger versus older than 40 years (mean difference of 0.332, 95% CI of difference -0.340 to 1.00, *P*=0.330) [Table 1].

**Table 1:** Patient gender- and age-based comparisons of esophageal Doppler monitoring versus pulmonary artery catheter bias characteristics

Patient group	N	Number of measurements	Bias ± S.D.^a^	95% C.I.^b^	S.E.M.^c^
Men (M) C.O.^d^	11	115	-1.01 ± 1.89	-4.79 to 2.77	0.177
Women (F) C.O.	4	40	-2.63 ± 2.16	-6.94 to 1.68	0.341
Difference (F vs M)^f^			-1.61	-2.33 to −0.902	*P*<0.001
Men (M) S.V.^e^	11	115	-9.12 ± 15.5	-40.1 to 21.9	1.52
Women (F) S.V.	4	40	-31.3 ± 20.7	-72.7 to 10.1	3.44
Difference (F vs M)^f^			-22.2	-15.7 to −28.6	*P*<0.001
Age <40 y/o	5	92	-1.30 ± 1.86	-5.02 to 2.42	0.194
Age 40 y/o	10	63	-1.63 ± 2.37	-6.37 to 3.11	0.298
Difference (<40 y/o vs 40 y/o)^f^			0.332	-0.340 to 1.00	*P*=0.330

a S.D. = Standard deviation,

b C.I. = Confidence interval,

c S.E.M. = Standard error of the mean,

d C.O. = Cardiac output,

e S.V. = Stroke volume,

f Student's t-test

In terms of endpoint of resuscitation assessment, mean serum lactic acid levels were significantly higher (3.89 ± 1.31) at the beginning of EDM monitoring periods as compared to the end of EDM monitoring periods (2.09 ± 0.946, *P*<0.006), supporting the efficacy of -EDM-directed clinical interventions [Table 2]. In addition, when compared with the initial values, the dosages of norepinephrine were significantly lower at the end of EDM monitoring periods [Table 2]. A similar comparison for neosynephrine use demonstrated no statistically significant difference [Table 2].

**Table 2:** Resuscitation parameters related to esophageal Doppler monitoring use

Duration of esophageal Doppler monitoring use (n = 15)		
12.4 ± 6.84 hours				
Median 12.5 hours			
Range 3 to 21.5 hours			
	**Beginning of resuscitation**	**End of resuscitation**	*P*-value*
Serum lactic acid level (n = 15)*	3.89 ± 1.31	2.09 ± 0.946	*P*=0.006†
Use of neosynephrine (n = 4)*	145 ± 103	56.8 ± 75.7	*P*=0.068
Use of norepinephrine (n = 8)*	6.13 ± 4.32	3.46 ± 3.69	*P*=0.049†

* Wilcoxon signed ranks test,

† Indicates statistical significance

## Discussion

Esophageal echo-Doppler is emerging as an alternative modality for noninvasive monitoring of critically ill patients in numerous clinical settings, with reports from obstetric, trauma, pediatric, colon-rectal, intensive care, cardiac surgery organ donation and emergency medicine literature.[3–8,10–12] Recently, the clinical efficacy of the EDM has been corroborated by two prospective, randomized, controlled trials.[1–2]

While other studies demonstrated findings similar to ours with regard to the lower cost of EDM use and requirement for fewer health care practitioners to place the EDM as compared to the PAC, the most striking advantage of the EDM appears to be the time to acquisition of continuous data.[11] In this study, not only did the CCO-PAC require significantly more health care personnel to place, but it took nearly 1 h from the beginning of the PAC placement procedure to continuous signal acquisition. In contrast, the EDM took an average of 12 min to place, resulting in immediate, continuous hemodynamic data. In critically ill patients, the time of 40-50 min to continuous data acquisition represents a clinically significant difference. We postulate that the delay to data acquisition in the CCO-PAC is due mainly to the duration of time needed for equipment calibration. In contrast, once optimal signal acquisition is accomplished with the EDM, no further calibration is needed.

Due to the variability in EDM monitoring time in this study, it is difficult to assess how much of the total resuscitation time was spent under direct EDM monitoring and how much under subsequent PAC monitoring. However, previous literature points out that approximately 75% of EDM-guided resuscitations are successfully completed within 6-8 h of the initiation of EDM monitoring and that resuscitations requiring longer monitoring periods may be better performed with the use of the PAC.[9]

This study examines EDM monitoring findings in a critically ill group of ICU patients, as evidenced by the APACHE II physiologic scores. All patients in this study were mechanically ventilated and a significant proportion required vasopressors (12/15, 75%). This study joins a growing number of clinical reports that support the usefulness of the EDM technology in ventilated, sedated critically ill patients who require significant hemodynamic support.[5,13–15] Our observations generally support the notion that the EDM can accurately demonstrate clinically useful trends in hemodynamic variables during resuscitation of critically ill patients.[4,11] The statistically significant improvement in lactic acid levels following EDM-guided resuscitation periods supports findings from other studies, which demonstrated that EDM-guided resuscitations and intraoperative interventions result in improved resuscitation endpoints.[1–2,9] Although our study does not directly demonstrate improvement in patient outcomes with EDM-guided resuscitation, others did demonstrate reductions in morbidity and resource consumption among patients resuscitated with EDM guidance.[1–2] Despite the documented clinical usefulness of hemodynamic monitoring using the EDM, the reader should be aware of the ‘imperfections’ in the EDM measurements, so that potentially better clinical decisions can be made with the help of this technology.

One previous study of pregnant women with preeclampsia demonstrated that the EDM consistently underestimated cardiac output in that population by approximately 40%.[7] Our data seem to support this gender-specific observation, with a notable measurement bias indicating significant overestimation in cardiac output and stroke volume in women who were monitored with the EDM as compared to the CCO-PAC. Although other studies show some bias when examining the EDM versus PAC in the overall population, no other studies specifically examined gender differences in this setting. We believe that this is a significant finding that confirms that of a prior study and clinicians should be aware of this potentially clinically significant measurement bias when treating critically ill female patients using EDM guidance. While EDM measurement bias has been attributed to numerous causes, some of the known contributing factors include operator inexperience (bias tends to decrease with operator experience); gender-related physiologic, rheologic and vascular differences; as well as alterations in vascular tone, as shown in one study of EDM monitoring of patients undergoing lumbar epidural analgesia.[16–18] In one study, 14 male patients underwent hemodynamic monitoring using both EDM and PAC before and after placement of lumbar epidural anesthesia. The baseline bias on the Bland-Altman plot in that study was nearly identical to the bias seen for male patients in our study.[17] However, following institution of lumbar epidural anesthesia, the EDM actually overestimated cardiac output relative to the PAC by approximately 0.51 liters/min.[17] Summary of major published series demonstrating measurement bias associated with EDM in various clinical setting use can be found in Table 3.

**Table 3:** Comparisons of esophageal Doppler monitoring-associated bias with pulmonary artery catheter cardiac output measurements in other published studies

Author (Reference)	Year (population)	Type of study of patients	Number	Population	Bias ± SD	Comment
Freund[16]	1987	Elective surgery patients under general anesthesia (versus PAC)^a^	23	23 men	0.16 ± 0.82 L/min	Correlation between thermodiltion and EDM increased with operator experience.
Lefrant *et al.*[18]	1998	Critically ill ICU^b^ patients (versus PAC)^a^	60	45 men		Showed that 12 insertions are needed for a trainee to become proficient in EDM placement. Bias tended to decrease with operator experience.
				15 women			
				Trainee-performed	-1.2 ± 3.2		
				Non-trainee	-0.1 ± 2.2	
Penny *et al.*[7]	2000	Women with preeclampsia (versus PAC)^a^	17	17 women	-2.0 ± 3.0 L/min	EDM more accurate in women >40 y/o.
Leather *et al.*[17]	2001	Men undergoing lumbar epidural anesthesia (versus PAC)^a^	14	Men pre epidural	-0.89 ± 0.89 L/min	Overestimatino of CO during lumbar epidural anesthesia as compared to PAC.
				Men post epidural	0.55 ± 1.88 L/min
Lafanechere *et al.*[6]	2006	Infrarenal aortic surgery (versus PAC)^a^	22	18 men		Bias between both methods was clinically acceptable, and limits of agreement were not significantly affected by aortic clamping.
				4 women		
				Pre aortic clamp	0.10 ± 0.73 L/min	
				During clamp	0.54 ± 1.05 L/min	
				Post clamp	0.18 ± 1.00 L/min	
Stawicki *et al.*[9]	2007	Critically ill SICU^c^ patients (versus PAC)^a^	39	26 men	0.02 ± 0.94 L/min	EDM tends to underestimate CO in the lower range, and tends to overestimate in the upper range of CO values relative to PAC. Note the minimal bias associated with extensive EDM operator experience.
				13 women		
Current study	2007	Critically ill study SICU^c^ patients (versus CCO-PAC)^d^	15	11 men	-1.01 ± 1.89 L/min	EDM demonstrated significantly more measurement bias in women than in men when compared to CCO-PAC.
				4 women	-2.63 ± 2.16 L/min	

a PAC = Pulmonary artery catheter,

b ICU = Intensive care unit,

c SICU = Surgical intensive care unit,

d CCO-PAC = Continuous cardiac output pulmonary artery catheter, EDM = Esophageal Doppler monitoring

While an apparent increase in EDM measurement accuracy was previously reported among women with preeclampsia who were >40 years old as compared to those who were <35 years old, there was insufficient evidence to determine whether this observation was due to chance or whether there was a real difference in EDM performance across the two age groups.[7] Our data does not confirm this finding, as we did not find significant differences in either bias or accuracy between patients ≥40 years old and those <40 years old. However, our study does not include sufficient number of women to perform a separate age-based comparison among female patients alone.

Despite measurement biases apparent in this and other studies, the EDM will likely continue to be a useful hemodynamic assessment tool. One must keep in mind that it is the intensivist's clinical judgment and not the monitoring equipment that will ultimately determine individual patient outcomes. Thus, knowing the equipment and its biases will allow practitioners to utilize the valuable clinical trends that the EDM provides and to use other adjunctive confirmatory laboratory and hemodynamic testing when necessary. Given the small overall sample size and comparison group size in this report, further validation of the above observations is warranted and the information presented should be regarded mostly as pilot data.

Limitations of this study include its small size, variability in EDM monitoring period, lack of PAC-only control group (and thus lack of direct modality comparison based on ‘monitoring equipment’-based interventions) and lack of patient outcome data. In addition, the patient group included in this study represents a convenience sample based on the availability of EDM-trained personnel. This study's strengths include its prospective nature, the demonstration of resuscitation endpoint improvement and decrease in vasopressor requirement during EDM monitoring periods and the large number of paired EDM-PAC data points allowing for meaningful measurement bias analysis.

## Conclusions

Existing literature studies support the effectiveness of the EDM technology in guiding patient resuscitations in a multitude of clinical settings. However, clinicians should be aware of the biases inherent to this technology and should consider these biases when caring for their patients. This is a second known study that described a significant negative bias in EDM measurements of cardiac output in women when compared to CCO-PAC. This study did not confirm the previously observed difference in EDM measurement accuracy and bias based on patient age.
